# Translational Phytomedicines against Cancer: Promise and Hurdles

**DOI:** 10.34172/apb.2023.023

**Published:** 2022-04-28

**Authors:** Aswathy R Devan, Bhagyalakshmi Nair, Lekshmi.R. Nath

**Affiliations:** Department of Pharmacognosy, Amrita School of Pharmacy, Amrita Vishwa Vidyapeetham, AIMS Health Science Campus, Kochi-682041, Kerala, India.

## Dear Editor,

 Cancer is the leading cause of death which evolves with the interaction of genetic, lifestyle and environmental factors. Sedentary behaviour, unhealthy food habits and physical inactivity are the major risk factor for cancer, which account for 70-90% of cases and also associated other lifestyle disorders.^1^ In 2020, GLOBOCAN estimated 19.3 million new cancer cases and near 10 million cancer-related death worldwide, in which breast cancer ranks as the most diagnosed and lung cancer remains as the deadliest.^2^ The current cancer therapy mainly involve surgical or radiation treatment followed by systemic drug therapy. Among the other treatment modalities, chemotherapy gained epochal recognition after the discovery of cytotoxic drugs during the Second World War. They further evolve as new anticancer molecules with promising effects. But, in the case of many available anticancer drugs, the factors such as drug-related adverse reactions, the emergence of multidrug resistance, and high cost affect the patient compliance and the majority of the patient can not adhere to the regimen.^3^ In this context, phytochemicals are getting much attention in cancer research due to their ability to target multiple molecular pathways of carcinogenesis without any toxicity. They are extensively being evaluated for chemoprevention; to reverse or prevent carcinogenesis, chemo sensitization; to enhance the sensitivity of cytotoxic drugs and chemotherapy; to treat cancer.

 The words ‘prevention is better than cure, quoted by a Dutch philosopher, Desiderius Erasmus in around 1500 (15-16^th^ century) is still relevant. Same is the case of cancer, it is prime to prevent this life-threatening disease rather than trying for other options like a treatment or sensitization strategy. Therefore, the concept of chemoprevention is getting much acceptance nowadays as the number of cancer cases are increasing day by day. More than 250 population-based studies reported a remarkable reduction of cancer occurrence after regular intake of fruits and vegetables.^4^ In 2002, WHO reported around 2.7 million deaths/year worldwide due to low intake of fruits and vegetables. More than 1000 chemo preventive phytochemicals have been identified in various preclinical studies. It was estimated that more than 100 important phytochemicals can be obtained from just one vegetable serving.^5^ The importance of vegetables and fruits in chemoprevention and the increasing number of cancer cases geared implementation of several international chemo preventive initiatives such as A Five-A-Day for better health program, Savor the spectrum, European prospective investigation of cancer and nutrition, Global strategy on dietary prevention of cancer, etc.^6^ Phytochemicals are an integral candidate in the prevention of cancer as they can be taken through a daily diet rich in fruits and vegetables or a supplement that can provide active phytochemical in the required daily dose. But this strategy will only work after proper validation of phytochemical in terms of efficacy, safety through well-designed clinical studies to achieve the desired protection against various cancer.

 Dietary phytochemicals elicit chemo preventive effects either by blocking carcinogenesis or suppressing the transformation of pre-neoplastic cells to neoplastic cells. Cancer blocking agents prevent metabolic activation of pro-carcinogens and boost up their detoxification, thereby inhibiting the initiation of carcinogenesis. Most of the compounds showed a significant effect in various preclinical studies. Sulforaphane, ellagic acid, and indole-3-carbinole are the important phytochemicals with cancer blocking action, whereas beta carotene, curcumin, EGCG, resveratrol, 6-gingerol, genistein, capsaicin, etc are reported to have cancer-suppressing action by induction of apoptosis and differentiation, inhibition of oncogene activity as well as scavenging free radical, etc.^7^ Several epidemiological studies suggested the cancer-preventing effect by regular intake of flavonoid rich food as well Phyto molecules mainly curcumin, quercetin (NCT01538316, NCT03476330), berberine (NCT03281096), sulforaphane (NCT03232138), EGCG (NCT02891538, NCT00917735), resveratrol (NCT00098969, NCT00578396), kaempferol, silibinin, luteolin, baicalein, etc via inhibition of pro-carcinogenic signaling that triggers the malignant transformation of cells.^8^ But, upon evaluating in an *in vivo* or clinical setting, majority of the mainstream phytochemicals failed to achieve an effective *in vivo*effect, which is apparently due to the associated PK issues. However, structural or nano analogs of phytochemicals are being extensively developed and evaluated for chemo preventive action.

 Many research studies revealed the potential of a large number of phytochemicals mainly flavonoids, polyphenols, alkaloids, terpenoids, carotenoids, saponins, and quinones to enhance the sensitivity of cytotoxic drugs against the cancer cells. This may promote a change from the conventional one drug-one target concept to combination therapies with safe-effective phytochemicals.^9^ Evidence of plant-based chemoprevention leads to more research findings of the molecular target of phytochemical to exert inhibitory effects on the cancer cell. These studies revealed the chemo sensitizing potential of phytochemical due to its ability to target relevant pathways involved in drug resistance with no or least toxicities. The emergence of multidrug resistance is posing a major obstacle in establishing an effective systemic drug therapy against cancer. Therefore, among the different strategies put forward to enhance the efficacy of conventional chemotherapeutics, phytochemical chemosensitizers are getting much acceptance nowadays because of their effectiveness and excellent safety. A detailed investigation of published literature found that phenolic phytochemicals such as curcumin, genistein, EGCG, quercetin, emodin, resveratrol are mostly reported with remarkable chemo sensitizing potential than other phytochemical classes.

 Most of the phytochemicals can simultaneously modulate multiple targets involved in chemoresistance. Diverse signaling events and multiple regulators of drug transport, apoptosis, cell survival, DNA repair, epithelial-mesenchymal transition are involved in the emergence of chemoresistance. The most figured out factor involved in drug resistance is the overexpression of efflux pumps (MDR1, p-gp, LRP, BCRP) which trigger the pumping out of drugs, thus unable to maintain cytotoxic concentration within the cell. Similarly, escape from cell death mechanism mainly apoptosis is another factor conferring resistance which is characterized by decreased levels of pro-apoptotic regulators such as p-53, Apaf-1, Bax and overexpression of anti-apoptotic factors such as Bcl-2, Bcl-xl, Mcl-1. Hypoxia, oxidative stress and inflammation can also contribute to MDR by overexpression of ROS, HIF-1 and NK-κB.^10^ These multi-targets of MDR rationally reveal the logic for phytochemical chemosensitization rather than available synthetic sensitizers such as verapamil or dexverapamil which act via modulating any one of the signaling events in MDR and thus, seems inadequate and ineffective.

 Curcumin enhances the cytotoxic potential of paclitaxel, docetaxel, gemcitabine, 5-FU, vinblastine, vincristine, and cisplatin by modulating multitude signaling such as NK-κB, Bcl2, Bax, Bak, surviving, VEGF, EGFR, IGF, MMP-9, P-gp etc.^11-13^ Furthermore, quercetin sensitizes doxorubicin, TRAIL, cisplatin, temozolomide by targeting resistance-conferring molecules such as HIF-α, surviving, MRP, p53, Akt, ERK, PKC.^14,15^ Our research team also reported the potential of kaempferol to sensitize sorafenib against resistant hepatocellular carcinoma cells. Molecular, as well as docking studies, confirmed that kaempferol work on resistant cancer cells by inhibiting mTOR, TGF-beta and P-gp and our team proposed that kaempferol can be validated as a potent yet safe mTOR inhibitor.^16,17^ As of 2013, approximately, there are more than six hundred published *in vitro* studies and around two hundred *in vivo* reports on the chemo sensitizing potential of phenolic compounds, which is significantly more than its reports for chemo preventive and chemotherapeutic effect.^18^

 Most of the phytochemicals that showed promising chemo sensitizing potential in preclinical evaluation have undergone clinical studies. Phytochemicals such as curcumin (NCT00295035, NCT00192842), green tea polyphenone E (NCT01116336, NCT00707252, NCT00088946), and genistein (NCT00376948, NCT00244933) were evaluated in clinical trial in combination with Erlotinib and gemcitabine against various cancers. Unfortunately, the molecules did not achieve the expected clinical effect. Curcumin itself can be the predominant example as it is the extensively evaluated phytochemical for chemo sensitizing potential with remarkable outcomes in most of the preclinical evaluations. But curcumin could not achieve the expected endpoint in most of the clinical trials, which point out the pharmacokinetic issues that make it unavailable in the systemic circulation. Likewise, resveratrol is also withdrawn from a clinical study, as it reported the development of cast nephropathy in 1/5^th^ of patients.^19^ Regardless of the immense chemo sensitizing property, the pharmacokinetic issues such as poor water solubility, rapid metabolism, short half-life and lack of toxicity studies hinder the clinical utility of phytochemicals. Several strategies are being evaluated to overcome the challenges with the application of nanotechnology. Resveratrol, curcumin, epigallocatechin, quercetin, rutin, betulinic aid, artemisinin, ginseng are the mainstream phytochemicals being converted to bioavailable nanoformulations such as a nanoparticle, liposome, phytosome, nano emulsion etc. But, still needs to be validated in well-designed clinical trials in terms of efficacy as well as safety.

 Plant based chemotherapeutics are the extensively investigated category of phytoresearch as 80 % of people worldwide rely on nature for primary health care and about 60% of currently used anticancer drugs are directly or indirectly derived from nature. Specifically, among the 240 chemotherapeutic agents approved in the last 40 years, 191 are derived from nature and the remaining 49 include the synthetic compounds derivatized from plant-based pharmacophore.^20^ A commonly available, as well as affordable phytochemical which can target major signaling molecules of cancer promotion, metastasis, and resistance with no toxic side effect, will be ideal for chemotherapy. Phytochemicals approved as chemotherapeutic agents can be majorly categorized as vinca alkaloids, epipodophyllotoxin, taxanes, and camptothecin derivatives.^21^ Vinca alkaloids and taxanes target tubulin and inhibit microtubule polymerization and thus leading to cell death. Podophyllotoxins target topoisomerase II to inhibit DNA synthesis of cancer cells whereas camptothecins target topoisomerase I to induce double-stranded breaks in DNA. Other phytochemical-derived anticancer agents are combretastatin A4, homoharringtonine, ingenol mebutate. Recently, FDA granted an orphan drug designation to uttroside B, a saponin from *Solanum nigrum* Linnfor the treatment of hepatocellular carcinoma and the compound showed 10 times more activity than the standard drug, sorafenib.^22-24^ Likewise, the phytochemicals such as curcumin (NCT03980509), resveratrol(NCT00256334, NCT01476592, NCT00433576, NCT01317953), artemisinin (NCT00764036, NCT03093129, NCT04098744) and ginseng (NCT00631852, NCT02603016) are under clinical investigation against various cancer types.

 Despite these tremendous research inputs, translational prominence of phytochemicals in oncology is considerably less as it warrants substantial evidence of better efficacy and least toxicity derived from well-designed clinical studies. Vinblastine is discovered in 1950 and got FDA approval after 15 years in 1965 for the treatment of leukemia and lymphoma. Similarly, for paclitaxel, it took around 25 years for approval as the drug for the treatment of ovarian cancer after its discovery in 1970. In addition to drug lag and associated expenses, the clinical translation of phytochemicals are also hindered due to complex extraction-synthesis procedure, difficulty in characterization and optimization, pharmacokinetic issues such as poor water solubility and bioavailability, rapid metabolism, formulation issues such as instability and route of administration.^25-27^

 Most phyto-research is undergoing a track change from direct phytochemical studies to synthetic or nano analogs to cover up the translational bridge. This could be the explanation for the fact that from 2010 to 2019, approximately 10 synthetic small molecules derived from a phytochemical pharmacophore are approved against cancer.^28^ Few examples are; the clinical limitations of paclitaxel especially poor bioavailability, drug-related toxicities and development of MDR is controlled to an extent with approved chemical or nano analogues such as; cabazitaxel for metastatic prostate cancer, paclitaxel poliglumex for glioblastoma multiforme, Abraxane (nanoparticle formulation of paclitaxel with improved bioavailability for refractory breast cancer and pancreatic cancer, EndoTAG-1 (Paclitaxel encapsulated in cationic lipid complex) for pancreatic cancer, as in the case of camptothecins, approved lipophilic analogues are cositecan, silatecan, gimetecan and diflomotecan and IMMUU-132, which is an antibody-drug conjugate of SN-38 (7-ethyl-10-hydroxycamptothecin) with orphan drug designation for the treatment of small cell lung cancer and pancreatic cancer.^29^

**Figure 1:**
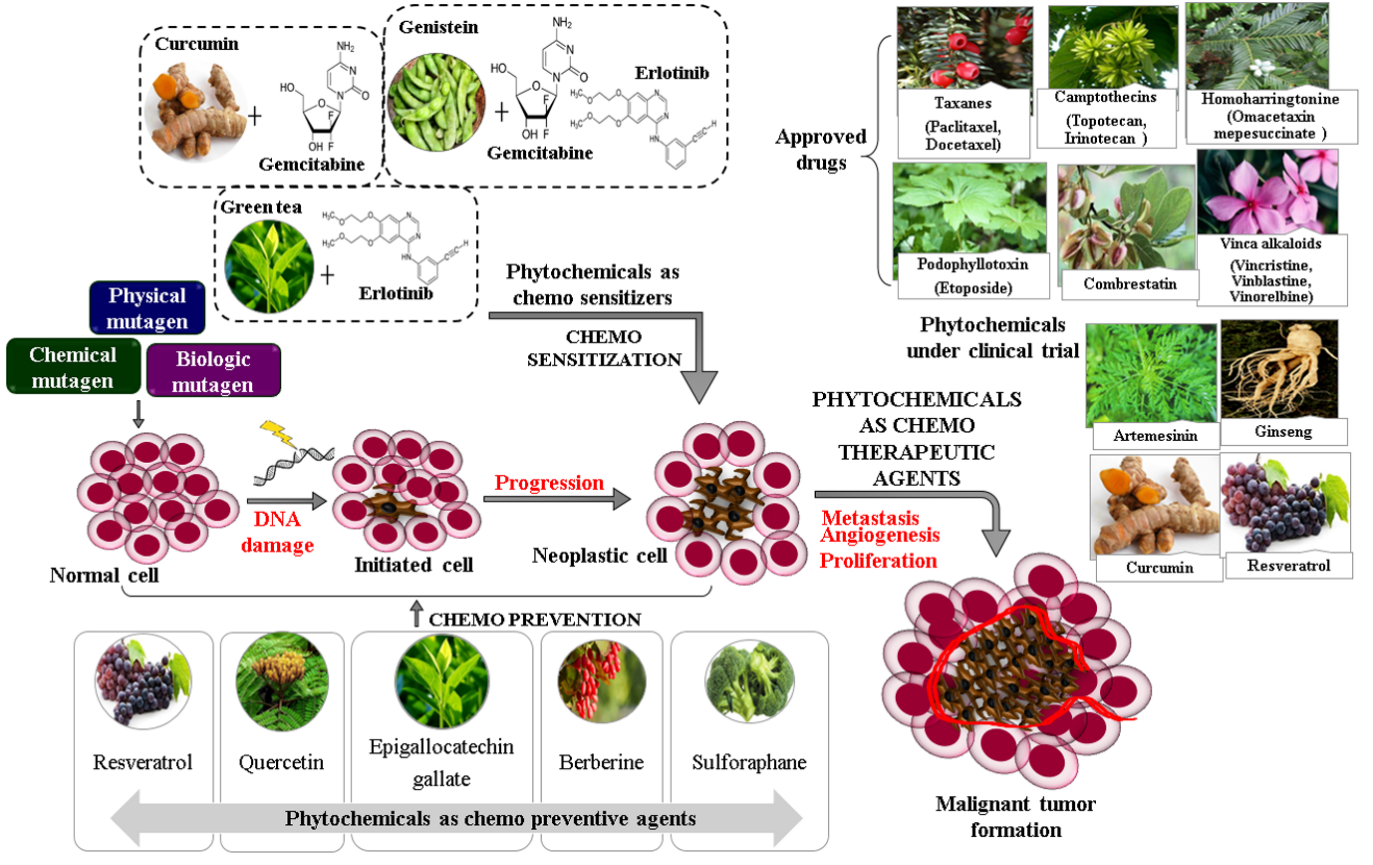
Phytochemicals as chemopreventive, chemosensitizing and chemotherapeutic agents

 Phytochemical are bestowed with enormous potential to act as chemo preventive, chemo sensitizing and chemotherapeutic agents by targeting a multitude of signaling involved in cancer initiation, promotion, progression as well as anticancer drug resistance (Figure 1). The multitargeting ability with excellent safety and obviously, affordability and availability make phytochemicals mainstream candidates of anticancer drug discovery. With advanced technology, phytochemicals are also undergoing ‘makeover’ to nano-phytomedicine or synthetic analogs without any conventional demerit of phytochemicals. Though the literature is expanding day by day, the clinically proven examples of plant-based anticancer agents are still a few as they have to prove having high efficacy in well-designed clinical trials rather than preclinical studies (Table 1). Therefore, the scientific community must focus to develop phytochemicals as safe-effective-available-affordable phytomedicine or supplements to fight against cancer.

**Table 1:** Phytomedicine has approved clinically available drugs as well as under status of clinical trials

**Phytochemicals approved as anticancer drugs**			
**Plant and Phytochemical category**	**Available approved drugs**	**Type of cancer**	**Mechanism of Action**
*Catharanthus roseus* Vinca alkaloids	Vincristine (Oncovin, Oncocrystine, Cytocristine) Vinblastine (Uniblastin, Cytoblastin) Vinorelbine (Vinotec, Relbovin)	Acute lymphocytic leukemia, chronic myeloid leukemia and Hodgkin and non-Hodgkin lymphoma Generalized Hodgkin’s disease, lymphocytic lymphoma, histiocytic lymphoma, mycosis fungoides, advanced carcinoma of the testis, Kaposi’s sarcoma Non-small cell lung cancer and metastatic breast cancer	Micro tubule damaging agents: Bind to tubulin and prevent its polymerization and assembly. Thus, cause mitotic spindle disruption.
*Taxus brevifolia* Taxanes	Paclitaxel (Altaxel, Mitotax, Oncotaxel) Docetaxel (Docecad, Docetere, Doxel)	AIDS-related Kaposi sarcoma, breast cancer, non-small cell lung cancer, ovarian cancer Breast cancer, non-small cell lung cancer, prostate cancer	Micro tubule damaging agents: Microtubule stabilization and inhibition of microtubule formation.
*Camptotheca acuminata* Camptothecins	Topotecan (Topotec, Cantop) Irinotecan (Camptosar, Irinotel, Irnocam)	Small cell lung cancer, cervical cancer Colon cancer, small cell lung cancer	Topoisomerase-I inhibitor: Binds to topoisomerase-I and stabilizes the formation of DNA-topoisomerase-I complex that leads to breakage of double stranded DNA.
*Podophyllum peltatum* Podophyllotoxin	Etoposide (Toposar, VePesid, Etopophos) Teniposide (Vumon, VM-26)	Small cell lung cancer, testicular cancer Acute lymphocytic leukaemia (children)	Topoisomerase-II inhibitor: Forms DNA and topoisomerase-II complex and inhibits DNA synthesis, blocks cell division and inhibits metaphase step of mitosis.
*Combretum caffrum* Combretastatin A4	Combretastatin A4 (Zybrestat)	Polypoidal choroidal vasculopathy, anaplastic thyroid cancers	Tubulin binding agent: Inhibits polymerization of tubulin causing disruption of the tumor endothelial cells lining the tumor vasculature.
*Cephalotaxus hainanensis* Homoharringtonine	Homoharringtonine/ Omacetaxine mepesuccinate (SYNRIBO)	Chronic myeloid leukemia	Binds to large ribosomal subunit, which affects chain elongation and prevents protein synthesis.
**Phytochemical as chemotherapeutics: Relevant clinical trial**			
**Phytochemical**	**Cancer type**	**Study details**	**Trial ID & phase**
Curcumin	Breast cancer	500 mg curcumin twice a day, immediately after meal, from the time surgical resection is scheduled until the night before surgical resection.	NCT03980509 Phase I
Resveratrol	Colon cancer	Patient receive resveratrol pills at a dose of 20 mg/d or 80 mg/d or 160 mg/d. All the patient receive 125 mg/d grape extract, before surgical resection.	NCT00256334 Phase I
	Neuroendocrine tumor	5 g/day of resveratrol orally, in two divided doses of 2.5 g each without a break in therapy for a total of three cycles.	NCT01476592
	Colon and rectal cancer	Patients receive oral resveratrol on days 1-8. Patients undergo colorectomy on day 9.	NCT00433576 Phase I
	Small cell lung carcinoma	Increasing doses of EGCG (400, 800, 1200, 1600 and	NCT01317953 Phase I
Artemisinin	Metastatic breast cancer	Add-on therapy with daily single oral doses of 100, 150 or 200 mg of artesunate for 4 weeks	NCT00764036 Phase I
	Colorectal cancer	Patients will receive 200 mg artesunate (Arinate®) per oral (PO) once daily (OD) for 14 days prior to their planned surgery and then be followed up for 5 years following surgery.	NCT03093129 Phase II
	Cervical intraepithelial neoplasia	Participants will receive three 5-day cycles of artesunate vaginal inserts, 200 mg/d, at week 0, week 2, week 4.	NCT04098744 Phase II
Ginseng	Breast cancer	four, 250 mg tablets daily 5-14 days prior to surgery	NCT00631852 Phase II
	Lung neoplasms, Breast neoplasms	Ginseng compound 2 tables each time by mouth, twice a day for 42 days.	NCT02603016
**Phytochemical as chemosensitizers: Relevant clinical trials**			
	**Chemotherapeutic**	**Cancer type**	**Trial ID & Phase**
Curcumin	Gemcitabine	Colon cancer	NCT00295035 Phase III
	Gemcitabine	Pancreatic cancer	NCT00192842 Phase II
Green tea polyphenon E	Erlotinib	Cancer of head and neck	NCT01116336 Phase I
	Erlotinib	Non-small cell lung cancer	NCT00707252 Phase I
	Erlotinib	Bladder cancer	NCT00088946 Phase II
Genistein	Gemcitabine, Erlotinib	Metastatic pancreatic cancer	NCT00376948 Phase II
	Gemcitabine	Stage IV breast cancer	NCT00244933 Phase II
**Phytochemical as chemo preventive agents: Relevant clinical trials**			
**Phytochemical**	**Cancer type**	**Study details**	**Trial ID & phase**
Resveratrol	Unspecified adult solid tumor	Studying the side effects and best dose of resveratrol in preventing cancer in healthy participants.	NCT00098969 Phase I
	Colon cancer	Dose level 1: 1 lb/day fresh red grapes Dose level 2: 2/3 lb/day fresh red grapes Dose level 3: 1/3 lb/day fresh red grapes	NCT00578396 Phase I
EGCG	Colon cancer	EGCG within 4-12 weeks of surgery and take EGCG 450 mg PO twice a day.	NCT02891538 Early Phase I
	Breast cancer	Two green tea extract capsules twice daily after breakfast and dinner for one year.	NCT00917735 Phase II
Quercetin	Prostate cancer	500 mg/d quercetin (+ vitamin C + folic acid + vitamin B3) over a period of six months; crossover design (6 month-periods): followed by genistein	NCT01538316
	Squamous cell carcinoma	Quercetin will be administered twice daily at an adjusted dose based on weight for a maximum total daily dose of 4000 mg/d	NCT03476330 Phase II
Berberine	Colorectal adenoma	Patients take the Berberine hydrochloride 300 mg tablet by mouth, 2 times a day with 3 years.	NCT03281096 Phase II
Sulforaphane	Lung cancer	Sulforaphane four tablets 2 times per day with breakfast and dinner each dose contains approximately 120 µmol of sulforaphane	NCT03232138 Phase II

## Acknowledgments

 We acknowledge the support of Dr. Reshma Ravindran Nair, Postdoctoral Research Associate, Wellcome Centre for Cell Biology, University of Edinburgh, United Kingdom for the language editing of the draft.

## Competing Interests

 The authors declare no conflict of interest.

## Ethical Approval

 Not applicable.
